# The Higher Temperature in the Areola Supports the Natural Progression of the Birth to Breastfeeding Continuum

**DOI:** 10.1371/journal.pone.0118774

**Published:** 2015-03-27

**Authors:** Vincenzo Zanardo, Gianluca Straface

**Affiliations:** Division of Perinatal Medicine, Policlinico Abano Terme, Piazza Colombo 1, 35031, Abano Terme, Italy; University of Alabama at Birmingham, UNITED STATES

## Abstract

Numerous functional features that promote the natural progression of the birth to breastfeeding continuum are concentrated in the human female’s areolar region. The aim of this study was to look more closely into the thermal characteristics of areola, which are said to regulate the local evaporation rate of odors and chemical signals that are uniquely important for the neonate’s ‘*breast crawl*’. A dermatological study of the areolae and corresponding intern breast quadrants was undertaken on the mothers of 70 consecutive, healthy, full-term breastfed infants. The study took place just after the births at the Policlinico Abano Terme, in Italy from January to February 2014. Temperature, pH and elasticity were assessed one day postpartum using the Soft Plus 5.5 (Callegari S.P.A., Parma, Italy). The mean areolar temperature was found to be significantly higher than the corresponding breast quadrant (34.60 ±1.40°C vs. 34.04 ±2.00°C, p<0.001) and the pH was also significantly higher (4.60±0.59 vs. 4.17±0.59, p<0.001). In contrast, the elasticity of the areolar was significantly lower (23.52±7.83 vs. 29.02±8.44%, p<0.003). Our findings show, for the first time, that the areolar region has a higher temperature than the surrounding breast skin, together with higher pH values and lower elasticity. We believe that the higher temperature of the areolar region may act as a thermal signal to guide the infant directly to the nipple and to the natural progression of the birth to breastfeeding continuum.

## INTRODUCTION

Within moments of a natural vaginal birth, without invasive birthing practices, newborns demonstrate innate feeding behaviors and instinctively seek out and ‘crawl’ toward the mother’s breast to attach themselves [1,2] their hands exploring and massaging the breast [3]. The functional steps that lead mammalian infants to seek out the nipple and attach themselves to it before proper sucking sets in are not fully understood [2], but it is known that early sucking behavior is easily upset by unfavorable conditions [4].

The human female’s areolar region is of special interest in this journey, because numerous features linked to the natural progression of the birth to breastfeeding continuum are concentrated in it [5] Apart from providing the infant with access to colostrum and milk, the nipple region provides chemical signals that appear to be particularly important to the neonate [6]. The attractiveness of maternal breast odors in the biological context of breastfeeding has a similar function to the role of ‘nipple search pheromone’ in guiding newborn mammalians to the nipple. Although maternal odors may not be as critical for nipple localization in the human species, they may nevertheless facilitate early breastfeeding attempts [7]. In addition to helping to guide the infant directly to the nipple, maternal breast odors affect other aspects of neonatal behavior that increase the probability of successful nipple grasping and feeding [8]. At the same time, they may help the infant to recognize its mother and, therefore, play a role in infant-mother bonding [9,10].

The areola region contains a dense accumulation of apocrine and sebaceous skin glands, which could contribute to producing attractive chemical signals or serve as odor fixatives that prolong the effectiveness of the odor [5]. In addition, the diffusion of odorous molecules may be enhanced by the relatively high surface temperature of the areola, due to the rich supply of blood vessels in this region [11,12]. There is some scientific evidence that describes how the infant behaves and responds to the odors that are naturally released by breasts of lactating women [13].

This study aimed to examine in greater detail how the infant locates the mother’s breast and to assess a number of previously overlooked dermatologic features. The primary objective of this study was to measure the temperature of the areola and the corresponding inside superior quadrant early after birth. Breast pH and elasticity were also assessed.

### Participants and Setting

Every woman who gave birth to a healthy full-term infant in the Department of Obstetrics and Gynaecology of the Policlinico Abano Terme, Abano Terme, Italy, between January and February 2014 was eligible to take part in the dermatological study of the areolae and corresponding intern breast quadrants. The study sample of 70 mother-infant dyads consisted of healthy breastfeeding women, without anomalies in the breast and, or, nipple anatomy or nipple dermatitis, who were at least 18-years-of-age, spoke Italian, had a low-risk pregnancy, and delivered a single healthy child of more than 37 weeks of gestation. The study was performed in accordance with the 1997 Declaration of Helsinki and the local institutional review board, the Ethics Commission of Policlinico Abano Terme, approved the study (Protocol N. 274/2013). If the inclusion criteria were fulfilled—the pregnancy and delivery were normal, the neonate was healthy and the mother intended to breastfeed—the mothers were asked to sign an informed consent form saying that they agreed to participate in the study.

The standard practice in our Department after an uneventful delivery is to place the infant on the mother’s chest immediately after birth and leave them there for 15 minutes while the midwife helps the mother to achieve the first suckling episode. The infants are then dried, receive umbilical care, and are weighed before their first bath. During the next two days, the newborns stay on the ward in the same room as their mothers, who are encouraged to feed them on demand with no more than a four-hour interval between feeds. The infants receive complementary formula if the midwife does not think they are receiving sufficient breast milk. After each feed, the mothers are simply advised to wipe their areolas and nipples with a scentless, humid gauze pad.

The participating mother-infant dyads were studied during the first 24 hours after delivery. The mothers were informed about our study methods, but were blind to the specific hypotheses being tested. Breastfeeding patterns were defined according to the World Health Organization’s classifications: exclusive breastfeeding was defined as the infant only receiving maternal milk, complementary breastfeeding was defined as offering breast milk and infant formulas and exclusive formula feeding was defined as only offering bottle-fed formula [14].

#### Data collection

Information about the pregnancy, labor, delivery, Apgar score, neonatal birth weight and first breastfeeding attempts were collected from the mother and from her medical records.

#### Areolar dermatological features

The mothers’ breasts were inspected by a midwife and a member of the study group on the first day postpartum between feedings, before assessing the areolae and inside superior quadrants using the Soft Plus 5.5 device (Callegari S.P.A., Parma, Italy) [15]. The following areola and quadrant surface variables were collected:

Temperature. Measurement principle: infrared measurement of external temperature. Field: 20°-40°C. Resolution: 0.1cu (conventional units). Precision: ±3%. Description: ABS plastic pen-type probe with a sensor on the tip. Operating conditions: temperature 15÷35°C and maximum relative uncondensed humidity 80%. Measurement method: the two red lights of the pen probe were focused until only one was clearly visible on the area of skin to be measured.

pH. Measurement principle: double celled electrode. Field: 2–12. Resolution: 0.1. Precision: ±1%. Description: rechargeable electrode. Operating conditions: temperature 15÷35°C and maximum relative uncondensed humidity 80%. Measurement method: the electrode was placed delicately on the skin without pressing.

Elasticity. Measurement principle: stress/deformation. Field: 0–50cu. Resolution: 1cu. Precision: ±10%. Description: ABS plastic pen-type probe with a sensor on the tip. Operating conditions: temperature 15÷35°C. Measurement method: the probe was placed at right angles to the skin.

### Statistical analysis

A descriptive analysis was used to construct a qualitative evaluation of the clinical data. Continuous data were expressed as means and standard deviations (SD). Categorical data were compared using Fisher's exact test, while continuous data were compared using the Student’s t-test. A p-value less than 0.05 was considered significant. Statistical analysis was performed using R 2.12 software.

## RESULTS

The maternal and neonatal anthropometrical and clinical characteristics are outlined in Table 1.

**Table 1:** Maternal and neonatal anthropometrical and clinical characteristics at birth.

Puerperae (n)	70
Age (years)	32.5 ± 5.1
Primiparae	18 (26%)
Weeks of pregnancy	40 (38–42)
Vaginal delivery	55 (79%)
Cesarean delivery	15 (21%)
Birth weight (kg)	3.475 (2.500–4.650)
Male/Female	33/37
Breastfeeding at discharge:	
Exclusive breastfeeding	**63** (90%)
Breastfeeding plus complementary formula	7(10%)
Exclusive formula feeding	0

Data are expressed as number (n) and percentage (%) or median (IQR, interquartile range).

The mothers’ mean age was approximately 33 years and about 20% underwent a caesarean section, with 60% of these being elective. They were breastfeeding exclusively when they were enrolled in the study. At discharge, most of the mothers were still breastfeeding exclusively, with seven (10%) giving their infants some formula milk. The infants’ mean body weight at discharge was 5.7% lower than their birth weight.

Table 2 shows the dermatologic features of the areola and corresponding inside breast quadrant.

**Table 2:** Dermatologic features of the areola and corresponding inside breast quadrant.

	Quadrant	Areola	
Puerperae (n = 70)	140	140	p
Temperature, C°	34.04±2.00	34.60±1.40	0.001
pH	4.17±0.59	4.60±0.59	0.0001
Elasticity (%)	29.02±8.44	23.52±7.83	0.003

Data are expressed as number (n) and percentage (%) or mean standard deviation (±SD).

The mean temperature values of the areola skin were significantly higher than the adjacent quadrant (34.60 ±1.40°C vs. 34.04 ±2.00°C, p<0.001).

The mean pH levels of the areola skin were also significantly higher then the adjacent quadrant (4.60±0.59 vs. 4.17±0.59, p<0.001).

In contrast, the mean elasticity values of the areola were significantly lower than the adjacent quadrant (23.52±7.83 vs. 29.02±8.44 cu, p<0.003).

## DISCUSSION

This study provides new information about some of the dermatologic properties of the areolar region in lactating mothers and some aspects of the ‘*breast crawl*’ phenomenon. Our findings show, for the first time, that the areolar region has a higher temperature than the surrounding breast skin, together with higher pH values and lower elasticity.

To the best of our knowledge, these dermatological features have never been examined using a scientific method during the period that breastfeeding is being established, nor have they been compared quantitatively with the surrounding quadrant skin. Higher areolar temperature, alone or combined with multiple sources of odorants and lipid fixatives, may serve as a heat-based communicative function and act as a thermal signal to guide the infant directly to the nipple.

Newborns can perceive the difference in temperature between the areola and the rest of the breast skin [16–18], just as they seem to be sensitive to, and exhibit a preference for, their mother’s scent [19]. In a series of studies, neonates were found to consistently prefer, and to move towards, the smell of their mothers’ breasts [20]. This could be due to a double action, both on the mouth sensitivity receptors and on the olfactory receptor neurons. In fact, lips and fingertips are often considered to be the areas with the highest concentrations of receptor cells [18]. Moreover, the areas of the brain that receive messages from touch receptors in the lips and hands are much larger than those receiving messages from less sensitive places and more brain power is spent interpreting touch sensations from the lips and fingers than from other areas [18].

This thermal characteristic may also play a role in regulating the local evaporation rate of odorants, thereby enhancing their effective role in stimulation. This is linked to olfactory learning, which is essential for neonatal behavioral adaptation in many mammals, including humans [19–21]. Birth and the first hours of life are crucial for olfactory learning. During this period the smells emanating from the mother and newborn interact with one other and animal studies have shown that there is an interaction in processing olfactory signals on both sides [21,22]. Thermal and olfactory signals may help the newborn to locate the nipple and attach to it, leading to the first sucking experience.

Although it is only small, the nipple-areolar region is densely supplied with varied **exocrine** and enlarged sebaceous skin glands, with small prominences that Morgagni called ‘‘tubercles” [2], coalescing sebaceous glands and miniature mammary acini [2,23]. Colostrum and milk are released from the nipple through the main lactiferous ducts and their intrinsic olfactory qualities and odorants reflect the mother’s diet, metabolism and genetic constitution [24]. These varied sources of substrates combine to create a composite, highly complex odor cocktail. The higher pH found in the areolar skin originates from this mixture and may indeed favor, or act as an odor fixative, to improve the stability of the olfactory complex that is formed on the surface of the areolar [25]. In addition, Haller’s sub areolar vascular plexus provides the areolar with a higher surface temperature than the nipple and the rest of the breast [11]. As we hypothesized earlier in this paper, this thermal characteristic may regulate the local evaporation rate of odorants, thereby enhancing their stimulating effectiveness. A vaginal delivery sets off a surge of catecholamine, which facilitates the newborn’s adaptation to extra uterine life and extensive crying [26]. It is interesting to note that the areola’s thermal feature is triggered by the crying infant [27], resulting in optimal conditions for odor release in anticipation of when the infant is offered the mother’s breast.

We presume that the characteristics of the areolar region that we report here have a biological significance. Firstly, the higher pH during early lactation may be linked to local epidermal and ductal protection from pathogens [28]. Secondly, lower areolar elasticity and greasy secretions may help to preserve the skin from the corrosive action of the infant’s saliva and from sucking-related stress [29]. Thirdly, skin gland secretions, infant saliva and lower elasticity may all be needed to create the hermetic seal that makes sucking effective [30].

Using bioengineering methods to assess nipple and breast skin features during the natural progression from birth to breastfeeding has not eliminated several study limitations. In particular, it was impossible to calculate the statistical power as there is no data in the literature regarding areolar pH, temperature or elasticity. More information about the ‘breast crawl’ phenomenon would be necessary to clarify other variables (i.e. maternal age, parity, if labor was induced, if and what type of analgesics were used, and the delivery route) that could influence mothers’ hormonal and neuroendocrinal response [31] and confound the final outcome. We did not observe the mothers’ attitude nor did we monitor their psycho-emotional and hormonal changes during the ‘breast crawl’ and early breastfeeding which could influence breast skin pH, temperature, and elasticity. These and the fact that there was no control group, can be considered other study limits.

### Conclusions

The functional characteristics of the human areola may fulfill multiple mechanical, protective and communicative functions. These include the infant’s ability to locate the nipple and grasp it with their lips, postpartal adaptation, energy conservation and the natural progression of the birth to breastfeeding continuum in the mother-infant dyad. Accordingly, we can hypothesize that the higher temperature of the areolar may facilitate sensory responses and the initial attempts by infants to ‘breast crawl’ while skin-to-skin with the mother. This then promotes the earlier onset of lactation in the mothers.
